# A possible regulatory link between Twist 1 and PPARγ gene regulation in 3T3-L1 adipocytes

**DOI:** 10.1186/s12944-016-0361-x

**Published:** 2016-11-08

**Authors:** Rui Ren, Zhufeng Chen, Xia Zhao, Tao Sun, Yuchao Zhang, Jie Chen, Sumei Lu, Wanshan Ma

**Affiliations:** 1 Department of Laboratory Medicine, Shandong Provincial Qianfoshan Hospital, Shandong University, Jinan, Shandong 250014 People’s Republic of China; 2 Department of Endocrinology, Qingdao Municipal Hospital, Qingdao, 266071 People’s Republic of China

**Keywords:** Twist 1, PPAR γ, Adipose tissue, Gene regulation

## Abstract

**Background:**

Peroxisome proliferator-activated receptor γ (PPARγ) is a critical gene that regulates the function of adipocytes. Therefore, studies on the molecular regulation mechanism of PPARγ are important to understand the function of adipose tissue. Twist 1 is another important functional gene in adipose tissue, and hundreds of genes are regulated by Twist 1. The aim of this study was to investigate the regulation of Twist 1 and PPARγ expression in 3T3-L1 mature adipocytes.

**Methods:**

We induced differentiation in 3T3-L1 preadipocytes and examined alterations in Twist 1 and PPARγ expression. We used the PPARγ agonist pioglitazone and the PPARγ antagonist T0070907 to investigate the effect of PPARγ on Twist 1 expression. In addition, we utilized retroviral interference and overexpression of Twist 1 to determine the effects of Twist 1 on PPARγ expression.

**Results:**

The expression levels of Twist 1 and PPARγ were induced during differentiation in 3T3-L1 adipocytes. Application of either a PPARγ agonist (pioglitazone) or antagonist (T0070907) influenced Twist 1 expression, with up-regulation of Twist 1 under pioglitazone (1 μM, 24 h) and down-regulation of Twist 1 under T0070907 (100 μM, 24 h) exposure. Furthermore, the retroviral interference of Twist 1 decreased the protein and mRNA expression of PPARγ, while Twist 1 overexpression had the opposite effect.

**Conclusions:**

There was a possible regulatory link between Twist 1 and PPARγ in 3T3-L1 mature adipocytes. This regulatory link enhanced the regulation of PPARγ and may be a functional mechanism of Twist 1 regulation of adipocyte physiology and pathology.

## Background

Adipose tissue is one of the most important functional organs in the human body and plays a critical role in human health. Adipose dysfunction is closely related to the occurrence and development of several diseases, including obesity, diabetes, insulin resistance and inflammation [1–3]. A number of genes are involved in the regulation of adipose function; however, the complex relationship between these genes has not been fully elucidated [4, 5]. Therefore, it is important to explore the regulation and function of key genes involved in adipose function.

Peroxisome proliferator-activated receptor γ (PPARγ) is a critical gene in the regulation of adipose function. Studies have shown that PPARγ is involved in several physiological functions of adipose, including adipocyte differentiation [6–8], adipokine secretion [9, 10], adipose tissue inflammation [11] and insulin sensitivity [12]. Therefore, investigating the regulation of PPARγ is important to further understand adipose function. Twist 1 is a well-conserved transcription factor that belongs to the basic helix-loop-helix (bHLH) family. Twist 1 has been shown to be important for the formation of adipose tissue, and recently, several studies have investigated the role of Twist 1 in adipose dysfunction. Twist 1 expression in subcutaneous adipose tissue and visceral adipose tissue is related to the pathogenesis of several metabolic syndromes, including obesity, inflammation and insulin resistance [13–15]. Therefore, PPARγ and Twist 1 are two genes with multiple similar functional roles in the regulation of adipose tissue.

However, it is unclear whether there is an interaction between Twist 1 and PPARγ in the functional regulation of adipose tissue. Recent studies have found that the function of Twist 1 in adipose tissue is closely related to PPAR family members. Twist 1 has been shown to be a PPAR delta-inducible, negative-feedback regulator of PGC-1 in brown fat metabolism, which has important implications for understanding metabolic control and metabolic diseases [16]. PPARγ has been shown to control the Twist 1-SMRT-GPS2 cascade, which is a regulatory transcriptional network involved in several functional processes in human adipocytes [17]. Previous studies from our laboratory also found evidence of Twist 1 and PPARγ regulation in 3T3-L1 preadipocytes. We showed that the retroviral interference of Twist 1 expression in 3T3-L1 preadipocytes enhances PPARγ expression after 4 days of hormone-induced differentiation [18]. These results indicate that the expression of Twist 1 and PPARγ are regulated by a complex interaction in adipose tissue. However, further studies are necessary to confirm the precise mechanism of this interaction.

The aim of this study was to explore whether changes in Twist 1 level influence PPARγ expression. We used retroviral interference and Twist 1 overexpression to determine the role of Twist 1 on the regulation of PPARγ. Also, we used a PPARγ inhibitor and agonist to investigate the effect of PPARγ on Twist 1 expression. We verified that PPARγ regulates Twist 1 expression. In addition, we showed that Twist 1 could also influence PPARγ expression positively. To our knowledge, the effect of Twist 1 on PPARγ expression is a novel finding. We have enriched the knowledge of the regulation mechanism of PPARγ, and these findings may explain the role of Twist 1 in adipocyte physiology and pathology.

## Methods

### Reagents

The 3T3-L1 mouse embryo fibroblasts and Dulbecco’s modified Eagle medium (DMEM) were obtained from ATCC (Rockefeller, Maryland, USA). Bovine serum and fetal bovine serum (FBS) were purchased from GIBCO (Invitrogen, CA, USA). The primary antibodies (anti-PPARγ and anti-Twist 1) were obtained from Abcam. The HRP-conjugated secondary antibody and the anti-β-actin primary antibody were purchased from ZSGB-BIO. The lentiviral vectors pGLV3/H1/GFP + Puro (LV3), LV4-EF1a-GFP/Puro (LV5), recombinant vectors LV3/Twist 1 shRNA and LV5/Twist 1 complementary DNA (cDNA) were all acquired from GenePharma (Shanghai, China). NE-PER™ Nuclear and Cytoplasmic Extraction Kit was purchased from Pierce (Thermo Scientific). SYBR^®^ Green Real-time PCR Master Mix (QPK-201 T) was purchased from TOYOBO. T0070907 and pioglitazone were both from Cayman Chemical (MI, USA). Insulin, isobutylmethylxanthine, cycloheximide (CHX), leupeptin, pepstatin A (L/P), proteasomes inhibitors (MG132) and the other reagents in this study were obtained from Sigma (Merck, German).

### Induction of differentiation and Oil Red O staining in 3T3-L1 preadipocytes

The 3T3-L1 preadipocytes were maintained in DMEM supplemented with 10 % bovine serum, 100 U/ml of penicillin and 100 mg of streptomycin at 37 °C in a humidified atmosphere composed of 95 % air and 5 % CO_2_. The induction of differentiation and the Oil Red O staining were conducted according to previously described methods [17].

### Twist 1 shRNA and Twist 1 cDNA lentiviral vector construction and packaging

The Twist 1 shRNA and Twist 1 cDNA lentiviral vectors were constructed and packaged by GenePharma. Twist 1 shRNA was generated based on previously described methods [17]. The recombinant vector, LV3/Twist 1 shRNA, and the negative control vector, LV3/NC, were identified using sequencing. For packaging, a lentiviral vector titer of 1x10^9^ TU/mL was used in the infection experiments.

The full Twist 1 cDNA sequence was synthesized using chemical methods. The Kozak protection sequence (GCCACC) and a NotI site (GCGGCCGC) were added to the 5′-end of the Twist 1 cDNA. A BamHI site (GGATCC) was added to the 3′-end of the Twist 1 cDNA. This complex was then inserted into the expression vector LV4-EF1a-GFP/Puro (LV5), which also served as a negative control in this study. A lentiviral vector titer of 1×10^9^ TU/ml of LV5/Twist 1 cDNA was used in the infection experiments.

### Lentiviral vector infection with LV3/Twist1 shRNA and LV5/Twist 1 cDNA

Mature adipocytes were infected with the lentiviral vectors (LV3/Twist1 shRNA, LV3/NC, LV5/Twist 1 cDNA and LV5/NC) 6 d after the induction of differentiation. The proportion of the lentiviral vector and high-glucose DMEM supplemented with 10 % FBS was 1:20. The cells were incubation for 8–12 h, and then, the infection medium was replaced with culture medium containing 10 % FBS and incubated for 48 h.

### Extraction of cytoplasmic and nuclear proteins

Cytoplasmic and nuclear proteins were extracted using a NE-PER™ Nuclear and Cytoplasmic Extraction Kit (Prod# 78833) according to the manufacturer’s instructions. Briefly, the cells were washed three times, harvested with 1 ml of PBS and centrifuged at 500xg for 5 min in microcentrifuge tubes. Ice-cold CER I buffer was added to homogenize the cells, and the homogenate was vigorously vortexed for 15 s and incubated for 10 min on ice. Next, ice-cold CER II buffer was added, and the mixture was vortexed for 5 s and incubated for 1 min. The mixture was centrifuged for 5 min at 16,000xg and the supernatant solution was transferred to a fresh microcentrifuge tube as the cytoplasmic protein fraction. The remaining pellet was suspended in ice-cold NER buffer and then vortexed for 40 min with 15-s breaks every 10 min. Finally, the samples were centrifuged at 16,000xg for 15 min to obtain the supernatant as the nuclear fraction.

### RNA extraction and real-time PCR detection

Total RNA was extracted using TRIzol (Invitrogen) according to conventional experimental procedure, and cDNA was synthesized based on standard protocols of RevertAid First Strand cDNA Synthesis kit (Thermo Scientific). Real-time PCR detection was conducted using the SYBR^®^ Green Real-Time PCR Master Mix Kit according to the manufacturer’s instructions. The PCR primers were designed using Primer 5.0 software, and the primer sequences has been shown in Table 1. The relative quantification of the target sequence was determined as the fold changes compared with GAPDH mRNA using the 2-ΔΔ Ct method. All reactions were conducted in triplicate.

**Table 1:** Sequence information on the primers used for RT-PCR

Genes	Sequences	Product size (bp)	Annealing temperature (°C)	Gene Bank
Mouse Twist 1	5′-CATGGCTAACGTGCGGGA-3′	124	60	NM_011658.2
	5′-CGCCAGTTTGAGGGTCTGAA-3′			
Mouse PPARγ	5′-GCTGACCCAATGGTTGCTGA-3′	181	60.5	NM_001127330.2
	5′-CTTTATCCCCACAGACTCGGC-3′			
GAPDH	5′-TGGCCTTCCGTGTTCCTAC-3′	178	59	NM_001289726.1
	5′-GAGTTGCTGTTGAAGTCGCA-3′			

### Western blot analysis

Western blotting was conducted according to previously described methods [18]. The membranes were incubated overnight at 4 °C with primary antibodies for anti-PPARγ (1:1,000), anti-Twist1 (1:50) and anti-β-actin (1:2500). The target molecular weights for Twist 1, PPARγ and β-actin were 21 kDa, 58 kDa and 43 kDa, respectively. The relative OD ratio was calculated using ImageJ software and normalized to β-actin. Data from three replicate experiments were analyzed.

### Statistical analyses

For Oil Red O staining, the data are presented as the OD values. ImageJ software was used to quantify the results from the western blotting experiment. The relative gray value of each band was calculated based on results of three replicates. All data are shown as the mean ± SD, and the data were analyzed using SPSS software package, version 17.0. T tests were used for comparisons between two groups. A one-way analysis of variance (ANOVA) was performed for comparisons between more than two groups, and Dixon’s *q* tests were used for further pairwise comparisons. *P* < 0.05 was considered significant.

## Results

### Twist 1 and PPARγ expression is induced during 3T3-L1 differentiation

**Fig. 1:**
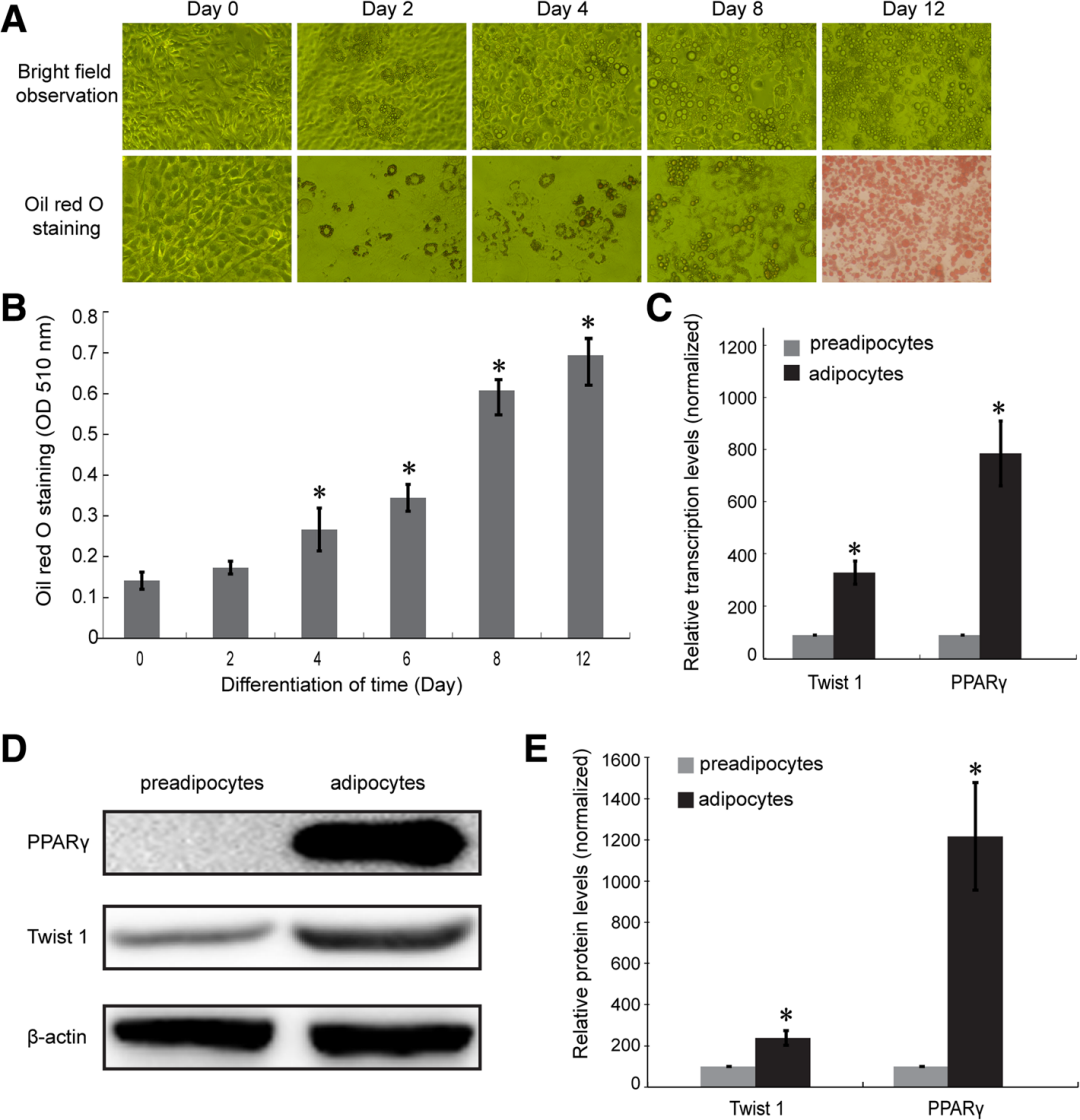
The induction of differentiation and the confirmation of Twist 1 and PPARγ expression in 3T3-L1 adipocytes. **a** The morphological changes observed in 3T3-L1 preadipocytes during differentiation under bright field and Oil Red O staining. **b** Quantification of lipids based on Oil Red O staining. **c** The transcription levels of Twist 1 and PPARγ were both elevated in adipocytes. **d** Twist 1 and PPARγ protein expression levels were upregulated during adipogenesis. **e** Quantification of the protein expression using ImageJ software

Differentiation was induced in 3T3-L1 preadipocytes according to previously described methods, and obvious morphological changes were observed during differentiation. As shown in Fig. 1a, the confluent 3T3-L1 preadipocytes exhibited a long shuttle shape without lipid droplets. During differentiation, a portion of the adipocytes began to accumulate lipids, which spread until most of the adipocytes were full of lipid droplets at day 8–12 after the initiation of induction. Lipid accumulation was quantified using Oil Red O staining (Fig. 1b), and significant differences in lipid content were observed 4 d after differentiation (*P < 0.05).

The mRNA and protein expression of Twist 1 and PPARγ in 3T3-L1 adipocytes were confirmed using real time PCR and western blotting, respectively. Figure 1c shows that there were significant increases in the mRNA levels of Twist 1 and PPARγ in the mature 3T3-L1 adipocytes (*P < 0.05). In addition, Twist 1 and PPARγ protein expression levels were significantly upregulated in mature 3T3-L1 adipocytes compared with the 3T3-L1 preadipocytes (Fig. 1d, e, *P < 0.05).

### Application of pioglitazone and T0070907 alters Twist 1 expression levels

**Fig. 2:**
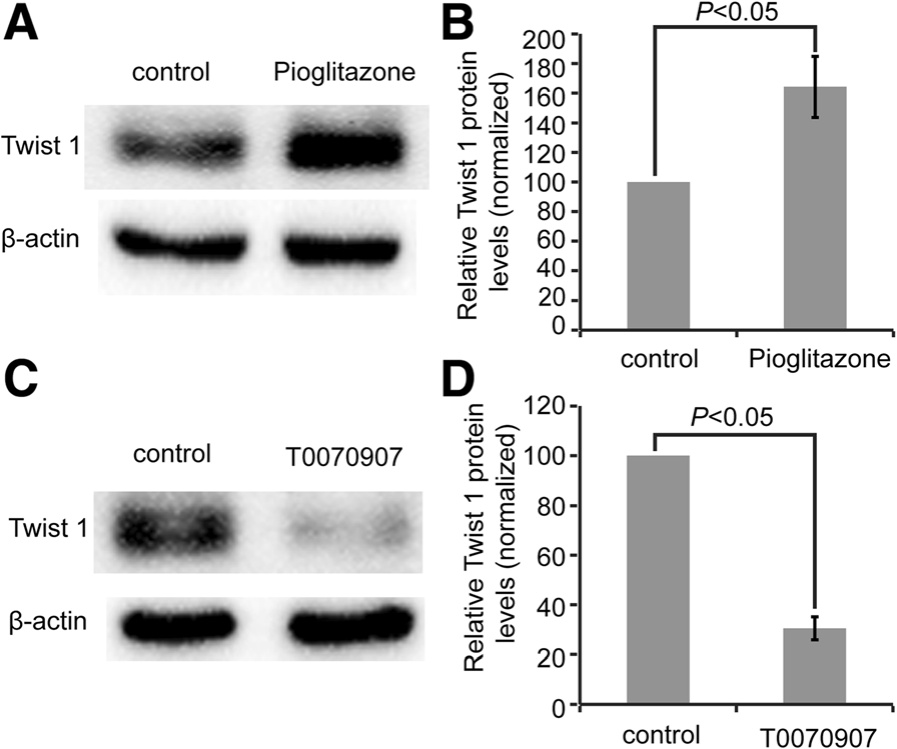
T0070907 and pioglitazone treatment changed Twist 1 expression. **a** The effect of pioglitazone (1 μM, 24 h) application on Twist 1 expression assessed by western blotting. **b** The relative quantification of Twist 1 expression after pioglitazone treatment. **c** T0070907 treatment (100 μM, 24 h) decreased Twist 1 expression, as detected by western blotting. **d** Quantification of the Twist 1 protein expression under T0070907 exposure using ImageJ software

To determine how PPARγ regulates Twist 1 expression, we added pioglitazone (1 μM, 24 h), a classic PPARγ agonist, and T0070907 (100 μM, 24 h), a common PPARγ inhibitor, into the culture medium of 3T3-L1 mature adipocytes and determined the protein expression of Twist 1. Taken the relative Twist 1 expression in both control group as 100 %, the expression level of Twist 1 in the pioglitazone (1 μM, 24 h) exposure group was significantly upregulated (164.3 ± 20.51 %, *P* < 0.05) (Fig. 2a, b), while that in the T0070907 (100 μM, 24 h) treatment group was (30.51 ± 4.62) %, significantly down-regulated (*P* < 0.05) (Fig. 2c, d).

### Retroviral interference or overexpression of Twist 1 regulates PPARγ expression by targeting protein synthesis

**Fig. 3:**
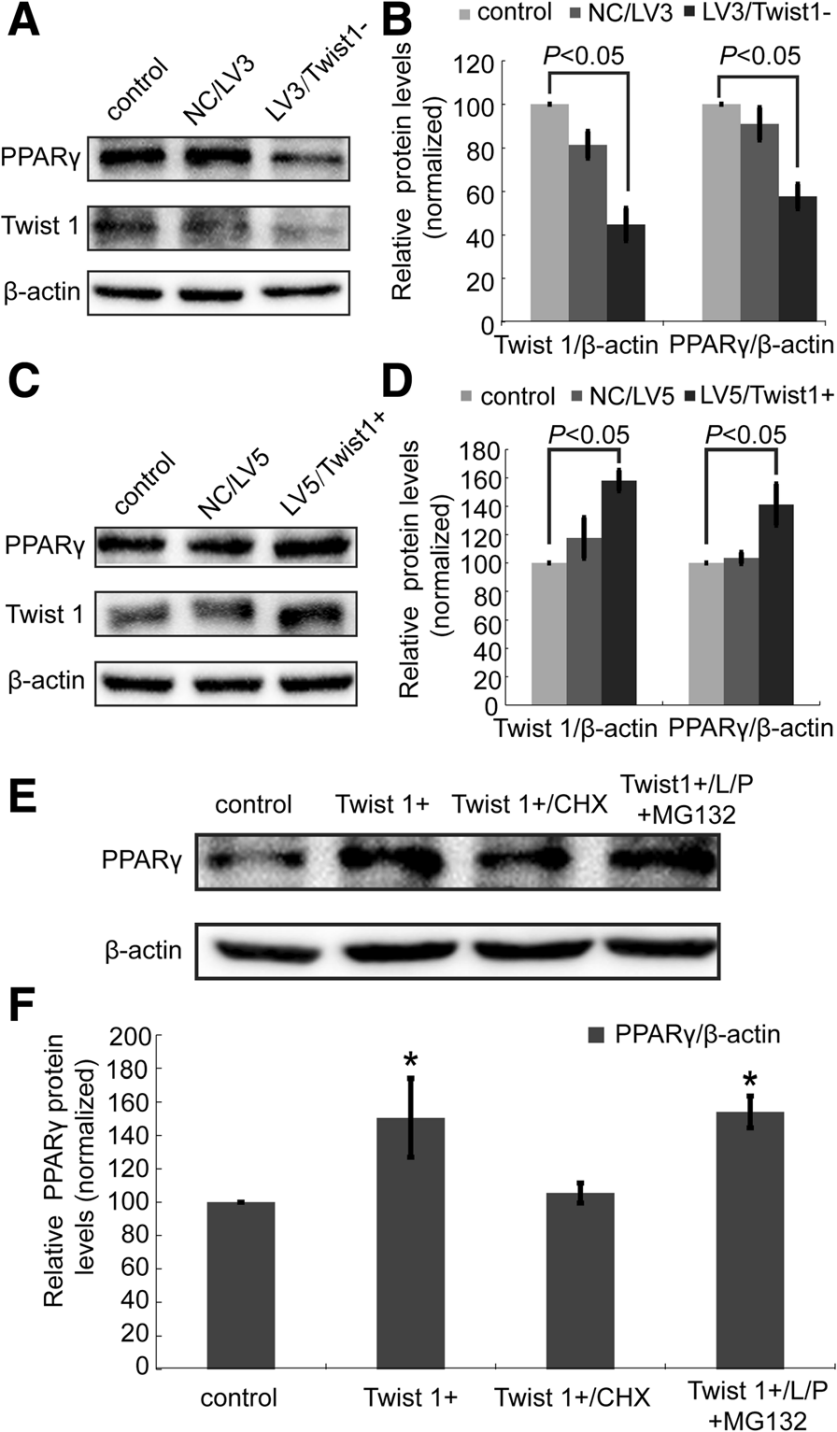
Retroviral interference or overexpression of Twist 1 positively regulated PPARγ expression by influencing PPARγ protein synthesis. **a** Twist 1 and PPARγ expression after shRNA targeted Twist 1 interference. **b** Quantification of the protein expression in (**a**) using ImageJ software. **c** Twist 1 and PPARγ expression after the overexpression of Twist 1 cDNA. **d** Quantification of the protein expression in (**c**) using ImageJ software. **e** Alterations in PPARγ protein synthesis and protein degradation after treatment with either CHX or L/P + MG132. **f** Quantification of the protein expression in (**e**) using ImageJ software

LV3/Twist1 shRNA (LV3/Twist 1- group) reduced Twist 1 expression to (44.80 ± 7.48) % compared with the control and LV3/NC group (*P* < 0.05), which suggest that retroviral interference was effective. Taken the relative PPARγ expression in control group as 100 %, the expression level of PPARγ in the LV3/Twist 1- group was (57.75 ± 5.66) %, significantly downregulated (*P* < 0.05) (Fig. 3a, b). While as shown in Fig. 3c, d, Twist 1 overexpression by the lentivirus vector LV4-EF1a-GFP/Puro (LV5) significantly increased Twist 1 expression to (158.12 ± 7.23) % in the LV5/Twist 1+ group (*P* < 0.05). Correspondingly, PPARγ expression was increased in the LV5/Twist 1+ group to (141.16 ± 14.55 %, *P* < 0.05).

Increased protein synthesis or decreased protein degradation might be responsible for the upregulation of PPARγ expression observed in our experiments. To confirm which pathway is involved, we incubated the 3T3-L1 adipocytes with either a protein synthesis inhibitor (CHX) or protease inhibitor (L/P) and proteasome inhibitor (MG132) 1 h before treatment with the LV5/Twist 1+ lentivirus vector. As shown in Fig. 3e, f, CHX abolished the LV5/Twist 1 + −induced increase in PPARγ expression (*P < 0.05), while L/P + MG132 application had no effect on the levels of PPARγ.

### PPARγ transcription was confirmed under retroviral interference or overexpression of Twist 1

**Fig. 4:**
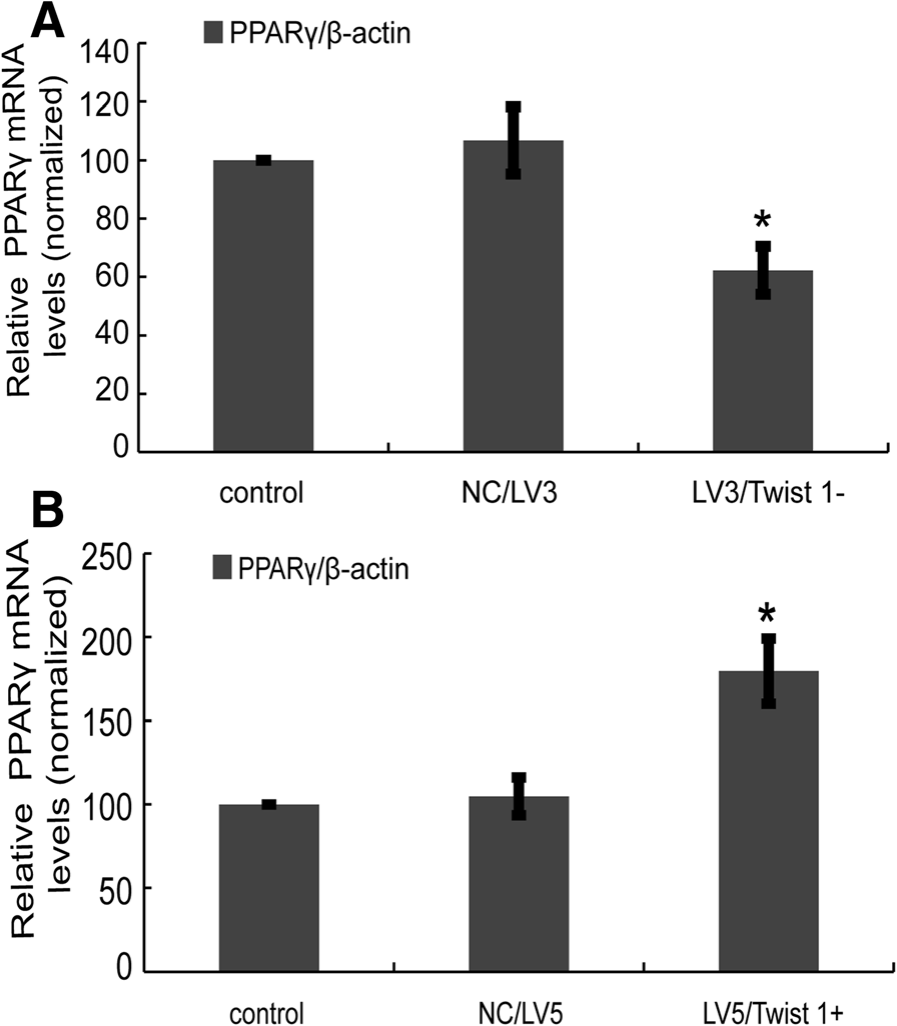
PPARγ transcription regulation after retroviral interference or overexpression of Twist 1. **a** LV3/Twist 1- treatment reduced the mRNA expression of PPARγ. **b** LV5/Twist 1+ treatment increased the mRNA expression of PPARγ

Given that Twist 1 regulates PPARγ expression by influencing protein synthesis, we next investigated transcriptional regulation of PPARγ to confirm this finding. As shown in Fig. 4a, retroviral interference of Twist 1 (LV3/Twist 1-) significantly decreased the mRNA expression of PPARγ transcription (62.2 ± 8.24 %) compared with the control group (set at 100 %). Twist 1 overexpression significantly increased PPARγ mRNA expression (179.53 ± 19.54 %; *P < 0.05) (Fig. 4b).

## Discussion

In the present study, we investigated the probable role of Twist 1 in the regulation of PPARγ expression. We found PPARγ and Twist 1 were both upregulated during adipogenesis. Application of pioglitazone increased Twist 1 expression and T0070907 exposure decreased Twist 1 expression level. Similarly, retroviral interference or overexpression of Twist 1 positively regulated PPARγ levels by influencing protein synthesis. To our knowledge, this is the first study to report that Twist 1 regulates PPARγ expression; however, PPARγ control of Twist 1 expression has been previously reported [17].

We have previously successfully induced mature and stable differentiation of adipocytes from preadipocytes [19]. In this study, we observed morphological changes that indicate adipogenesis (including the appearance of lipids) from day 4 after the induction of differentiation. PPARγ regulates adipocyte differentiation, and PPARγ upregulation is necessary and sufficient for adipocyte formation [20]. We detected PPARγ upregulation in adipocytes during differentiation, which is consistent with previous reports [17, 18, 21]. In addition, in this study, we showed that Twist 1 was upregulated during adipogenesis, which has been confirmed in our previous studies [18]. Overexpression of Twist 1 has also been shown to enhance the capacity of purified human bone marrow-derived mesenchymal stromal/stem cells to undergo adipogenesis [22]. Similarly, our results suggest that Twist 1 has a critical function in adipocyte differentiation. These preliminary results suggested that maybe there are some cross-functions for Twist 1 and PPARγ in adipocytes, indicated the possibility of probable regulation between Twist 1 and PPARγ. Further studies about the regulatory link between Twist 1 and PPARγ were necessary and meaningful.

The PPARγ antagonist T0070907 has been widely used to inhibit PPARγ activity [23–25]. In this study, we proved that Twist 1 expression of 3T3-L1 adipocytes was downregulated under T0070907, which indicates that PPARγ downregulation inhibits Twist 1 expression. Several studies have also used the PPARγ agonist pioglitazone to investigate the function of PPARγ [26]. The positive regulation between PPARγ and Twist 1 expression levels was also observed after pioglitazone treatment in the current study, with obvious up-regulation of Twist 1 in pioglitazone exposure group. This finding suggests that PPARγ positively controls Twist 1 expression. Similarly, studies in diabetic obese patients found that pioglitazone treatment increased Twist 1 expression in adipose tissue and showed that PPARγ controlled Twist 1 expression through the Twist 1-SMRT-GPS2 cascade, which is consistent with our findings [17].

We used retroviral interference or overexpression of Twist 1 to control Twist 1 expression. The results showed that Twist 1 downregulation significantly reduced PPARγ expression. Conversely, Twist 1 overexpression significantly increased PPARγ expression. These results indicate that alterations in Twist 1 expression positively regulate PPARγ, which has not been shown previously. As an important member of the bHLH family of transcription factors, hundreds of genes are regulated by Twist 1, including miRNAs [27, 28] and genes related to the epithelial-to-mesenchymal transition (EMT) in carcinomas [29, 30]. In addition, Hes4 is a novel bHLH transcription factor that has been shown to be regulated by Twist 1 and antagonize the function of Twist 1 in regulating lineage commitment of bone marrow stromal/stem cells [31]. In this study, we suggest that PPARγ is also under the regulation of Twist 1.

Two opposite processes may be responsible for the observed changes in protein expression: protein synthesis and protein degradation. An increase in protein expression may result from increased protein synthesis or decreased protein degradation. Therefore, we used CHX and L/P + MG132 to determine whether the regulation of PPARγ by Twist 1 was a result of changes in protein synthesis or degradation. Our results confirmed that the retroviral interference or overexpression of Twist 1 altered the protein synthesis, and not the protein degradation, of PPARγ, which was further confirmed by assessing the transcriptional regulation of PPARγ.

It is worth noting that, there seems to be some discrepancy between the results of our previous report [18] and the current results for the regulation relationship between Twist 1 and PPARγ expression. In our previous work, Twist 1 lentivirus interference increased PPAR gamma expression in the day 4^th^ differentiation induction cells, while a downregulation of PPARγ was reported under Twist 1 lentivirus interference in the current study. In fact, the research material is quite different between the present study and the previous work [18], which should be responsible for the main inconsistence. In our previous work, we infected Twist 1 interference lentivirus to the 3T3-L1 preadipocytes, and found an increase in PPARγ expression at day 4^th^ differentiation induction under interference of Twist 1. However, in the present study, mature adipocytes were our research objects, and Twist 1 interference lentivirus was infected to 3T3-L1 mature adipocytes after 12–14 days differentiation induction. As we know, adipogenesis process was a complex process regulated by multi-genes, adipocytes located in the day 4^th^ differentiation induction was not yet stable, and was a form of transitional cell phase, which means lots of instability and complexity. It can’t be excluded that the increase of PPARγ expression at day 4^th^ differentiation induction may not be the direct effect of Twist 1. But mature adipocytes experienced after 12–14 days differentiation induction were stable, without these above concerns. Further, in the present study, we introduced Twist 1 overexpression and PPAR gamma inhibitor to explore the full regulation relationship between Twist 1 and PPAR gamma, which can reflect the relationship of both genes from different levels.

In summary, in this study, we reported a possible regulatory link between Twist 1 and PPARγ, especially the probable role of Twist 1 in regulating PPARγ expression. PPARγ has been shown to control the Twist 1-SMRT-GPS2 cascade in human adipocytes [17]; however, our results suggest that an interaction between Twist 1 and PPARγ probably exists in 3T3-L1 adipocytes. This regulatory link significantly enriched the gene regulation mechanism of PPARγ and may explain the role of Twist 1 in adipocyte physiology and pathology.

Although not used in this study, a ChIP assay would definitively show the role of Twist 1 in regulating PPARγ. However, our results provide evidence of a regulatory link between Twist 1 and PPARγ, which indicate a possible mechanism of Twist 1 in adipocytes function. Further studies are necessary to fully elucidate the relationship between PPARγ and Twist 1 in adipocyte function.

## Conclusion

In this study, a possible regulatory link between Twist 1 and PPARγ in 3T3-L1 mature adipocytes was reported. This regulatory link enriched the regulation mechanism of PPARγ and may also be critical mechanisms involved in Twist 1 functions in adipocyte physiology and pathology. This study provides theoretical basis for further studies about the role and mechanism of PPARγ and Twist 1 in adipocytes.
